# Validation and optimization of the diatom L/D ratio as a diagnostic marker for drowning

**DOI:** 10.1007/s00414-023-02970-x

**Published:** 2023-03-04

**Authors:** Dominik Hagen, Stefan Pittner, Jian Zhao, Astrid Obermayer, Walter Stoiber, Peter Steinbacher, Fabio C. Monticelli, Walther Gotsmy

**Affiliations:** 1 Department of Ecology and Evolution, Paris-Lodron University of Salzburg, Salzburg, Austria; 2 Department of Forensic Medicine and Forensic Psychiatry, Paris-Lodron University of Salzburg, Salzburg, Austria; 3 Guangzhou Forensic Science Institute & Key Laboratory of Forensic Pathology, Ministry of Public Security, Guangzhou, China

**Keywords:** Diatoms, Drowning, L/D value, SEM

## Abstract

If a dead body is discovered in water, it nearly always raises the question about the cause of death, often associated with the persistent problem to differentiate between a drowning incident and post-mortem immersion. In numerous cases, a reliable confirmation of death by drowning is often only possible by a combination of diagnoses obtained from autopsy and additional investigations. As to the latter, the use of diatoms has been suggested (and debated) since decades. Based on the consideration that diatoms are present in almost every natural waterbody and are unavoidably incorporated when water is inhaled, their presence in the lung and other tissues can provide evidence of drowning. However, the traditional diatom test methods are still subject of controversial discussion and suspected of erroneous outcome, predominantly through contamination. A promising alternative to minimize the risk of erroneous outcome seems to be disclosed by the recently suggested MD-VF-Auto SEM technique. Especially the establishment of a new diagnostic marker (L/D ratio), which represents the factorial proportion between the diatom concentration in lung tissue and the drowning medium, allows for clearer distinction of drowning and post-mortal immersion and is largely robust to contamination. However, this highly elaborated technique requires specific devices which are frequently unavailable. We therefore developed a modified method of SEM-based diatom testing to enable the use on more routinely available equipment. Process steps such as digestion, filtration, and image acquisition were thoroughly broken down, optimized, and ultimately validated in five confirmed drowning cases. Taking certain limitations into consideration, L/D ratio analysis provided promising results, even in cases of advanced decomposition. We conclude that our modified protocol indeed opens a way for a broader use of the method in forensic drowning investigation.

**Supplementary Information:**

The online version contains supplementary material available at 10.1007/s00414-023-02970-x.

## Introduction

The diagnosis of drowning is a very difficult, and yet crucial aspect in forensic routine. Although there are numerous known macroscopic and microscopic indicators of drowning, most of them are insufficiently specific, only transiently applicable, and/or suffer from significant limitations depending upon injury, drowning medium, and/or post-mortem interval (PMI) [1, 2]. Especially in the case of advanced decomposition, conventional methods have limited scope. In search of a method that can provide reliable and unambiguous evidence, one repeatedly comes across the examination of organs for diatoms. These microscopic algae, present in almost every natural waterbody, are assumed to be incorporated by inhalation of the drowning medium during the drowning process and to (at least partially) pass the alveolo-capillary membrane, thus reaching distinct organs through distribution via the bloodstream [3]. If the examination of organs distant to the lungs, like the liver, kidney, or bone marrow, exposes a certain amount of diatoms, this can be regarded as supportive evidence for drowning. The previous methodology of the diatom test included the digestion of biological tissues with strong acids, followed by purification and deacidification steps, and lastly the assessment of the acid-stable silica diatom frustules via light microscopy [4].

Over the years, various studies repeatedly confirmed or criticized the supportive evidence of the diatom test, not least due to its vulnerability for contamination effects and false positive results in non-drowning cases [5]. Especially the examination of diatoms in lung tissue leaves this subject in controversial discussion, as diatoms are presumed to infiltrate the lungs post-mortem during the submersion period [5, 6].

A recently suggested method, the microwave digestion–vacuum filtration–automated scanning electron microscopy technique (MD-VF-Auto SEM), appears to be a promising new development in the field of diatom examination to minimize erroneous results from contamination and diatom loss during centrifugation steps. Combining diatom-sensitive microwave digestion and membrane filtration with automated scanning electron microscopy, this method achieves a remarkable quality of diatom recovery and ensures qualitative and quantitative examination at high resolution [7]. The additional establishment of a diagnostic marker (L/D ratio), which represents the proportion between the diatom concentration in 1g of lung tissue and 1ml of drowning medium, allows clearer distinction between drowning and post-mortal immersion, as an active aspiration of fluid in the case of drowning results in a relatively higher concentration of diatoms in the lung tissue than in the drowning medium (L/D ratio >1), whereas in the case of post-mortal immersion, the diatom concentration in lung tissue can at most reach equality with the concentration of the drowning medium (L/D ratio ≤ 1) [8]. Despite these advantages, this highly elaborated technique impedes routine application by requiring expensive “high-tech” devices including automated SEM for reliable diatom identification and counting, which are frequently unavailable.

In order to enable routine application on existing equipment, process steps as digestion, filtration, and image acquisition were thoroughly broken down, optimized, and ultimately validated in confirmed drowning cases, without detracting the method’s reliability and precision.

## Material and methods

### Sample collection

All tissue samples analyzed in the present study were collected with thoroughly cleaned instruments during routine autopsies at the Department of Legal Medicine of the University of Salzburg. For each case, approximately 10 g of lung tissue (left superior lobe) was removed and preserved at −20°C until further investigations. Additionally, 10 g of liver and kidney tissue was collected and stored under the same conditions to test diatom presence in peripheral tissues.

Water samples and putative drowning media (required for comparison, respectively L/D calculations) were collected in clean plastic bottles and sampled with caution, to avoid extraction close to the surface or close to benthic layers (minimum distance 20 cm, if possible).

### Control samples for protocol optimization

Lung tissue samples of three confirmed drowning cases served as controls (A, B, C) to evaluate, modify, and optimize the digestion procedure with nitric acid and hydrogen peroxide. In addition, these samples were tested for regular diatom dispersion and the correlation between diatom quantity and investigated tissue mass by assessing the number of diatoms from lung tissue samples of different weight. This was particularly important to enable adjustment of the tissue mass (dilution) in the case of membrane clogging or diatom-overload on the membrane, or to enhance digestive capability.

SEM image acquisition procedures were tested and validated at three dilutions (1:1, 1:2, 1:5) of diatom-rich water samples from a local pond.

### Study cases

Five autopsy cases (three males, two females, all found during spring/summer) were selected to validate the adapted processing conditions and to perform L/D ratio calculations. Corresponding drowning media was collected by the police upon discovery of the bodies and stored in dark environment at 4°C until further analysis.

Four of the cases presented distinct classical drowning signs, such as emphysema aquosum, foamy liquid in the airways, splenic anemia, and/or liquid in the sphenoid sinuses (Svechnikov’s sign), and thus were diagnosed as drowning cases. By contrast, the autopsy of case 4, which was in a state of advanced decomposition, solely exhibited liquid in the sphenoid sinuses and drowning was only presumed. For each case, 0.5–1.0 g lung tissue and 10 ml putative drowning medium were processed and digested under previously established conditions (see the “Acid digestion and filtration” section) and analyzed via SEM (see the “SEM analysis and image acquisition” section) to enable calculation of respective L/D ratios. Notably, the putative drowning medium of case 5 contained a significant amount of debris (plant particles), but was also processed as described. Study case data are presented in Table 1.

**Table 1:** Demographic data and autopsy findings of five potential drowning cases. *EA* emphysema aquosum, *FL* foamy liquid in air passages, *LSC* liquid stomach content, *SA* splenic anemia, *SS* Svechnikov’s sign

Case no.	Gender	Age	Season	Water body	Drowning signs	Additional findings
1	Male	70	Spring	River	EA, LSC, SS	-
2	Female	73	Summer	River	EA, SS, SA	-
3	Female	75	Spring	River	EA, FL	-
4	Male	53	Spring	River	SS	Advanced decomposition
5	Male	80	Summer	Pond	FL, SA, SS,	Debris in the drowning medium

### Contamination tests

To rule out false positive tests due to diatom-containing chemicals and contamination effects during sample processing, digestion reagents (nitric acid, hydrogen peroxide) and all cleaning and rinsing components (ultrapure water, tap water, ethanol) were filtrated onto acid-stable membranes and separately investigated for diatom content via SEM. In addition, a diatom-rich water sample was subjected to an evaporation test to rule out possible diatom loss and/or cross-contamination of samples during the digestion process. For this purpose, the digestion tube was fitted with a membrane underneath its cap. All reagents and other liquids, just as the evaporation membrane, were found entirely free of diatoms (see Supplements 1).

### Acid digestion and filtration

**Fig. 1:**
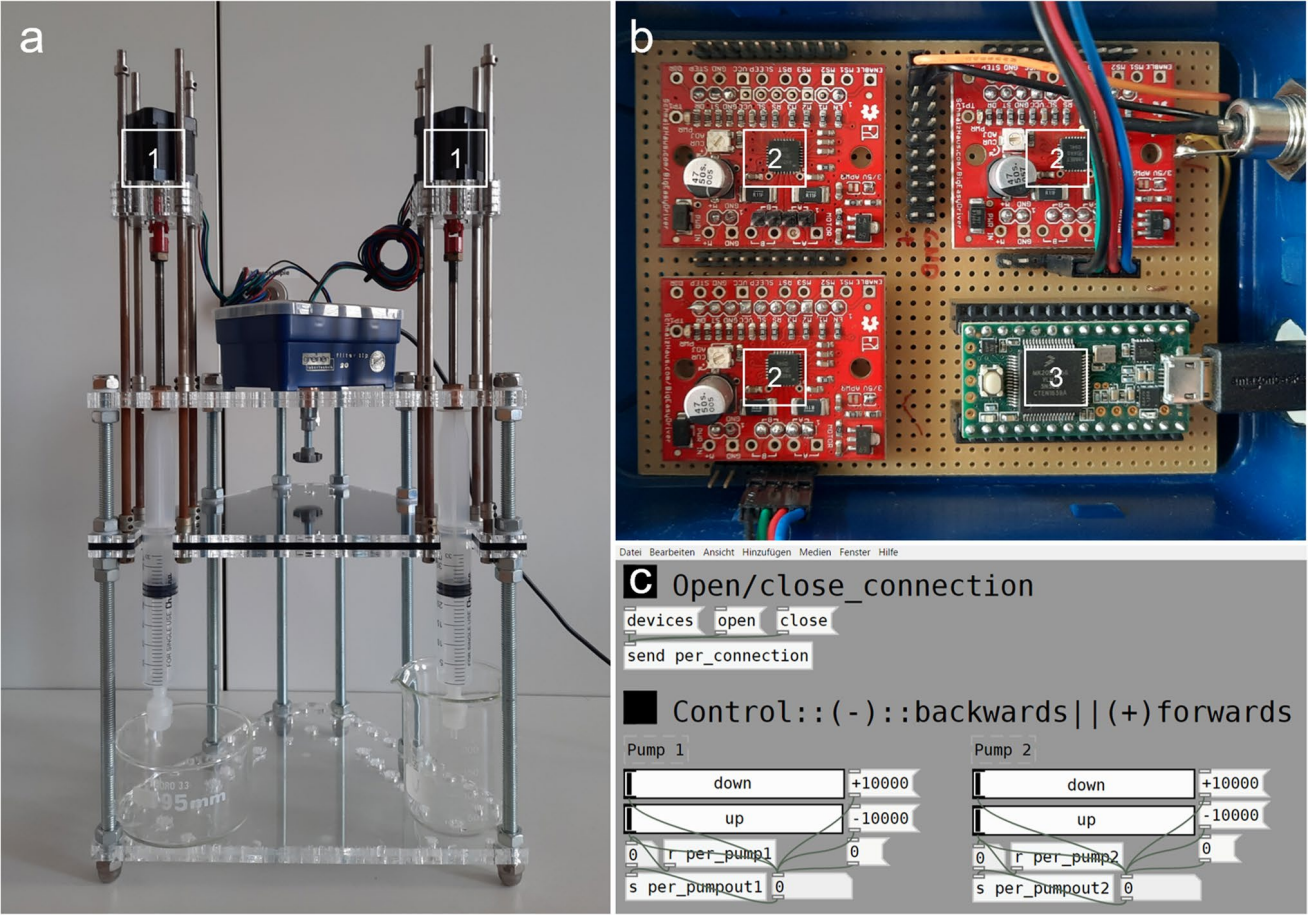
Filtration apparatus; **A** syringe-pump-system with stepper motors (1), **B** chip-setup with Big Easy drivers (2) and teensy microcontroller (3), and **C** operating software (PurrData); f**iltration time: approx. 17 ml/min (7500 rpm)**

Overall preparation was conducted under sterile conditions and high safety precautions. All samples (i.e., study case tissues, control tissues, drowning media, and water controls) were transferred into 50-ml screw cap plastic tubes (Greiner) and as an essential safety measure, tube caps were provided with small perforation holes to enable gas emission. Samples were then treated with a digestive solution of nitric acid (HNO_3_ 65%, CarlRoth) and hydrogen peroxide (H_2_O_2_ 30%, CarlRoth) while being heated in a water bath of 100°C until the solutions turned clear (at least for 2h) and afterwards left at room temperature for cooling. Instead of conventional deacidification by repetitive centrifugation and replacement of the supernatant, samples were then directly filtrated through acid-stable polyvinylidenfluoride (PVDF) membrane filters (Ø = 1.0 cm, pore size 0.45 μm) with a custom-built syringe pump system comprising NEMA 17 stepper motors linked to A4983 Big Easy driver chips and a PurrData-software-operated Teensy 3.2 microcontroller (Fig. 1). Membranes were subsequently deacidified with ultrapure water, desiccated with pure ethanol, and air dried at 40°C. As the digestive solution of lung tissue can contain relevant amounts of organic residues which potentially clog the membrane filters, provision was made to ascertain best conditions of organic matter digestion. Therefore, three different volumes of the nitric acid–hydrogen peroxide medium (5 ml, 10 ml, 15 ml) were each applied to 0.5 g, 1.0 g, and 1.5 g lung tissue control samples to test their digestive capability. In more detail, three samples of each weight were respectively digested with 4 ml HNO_3_ + 1 ml H_2_O_2_, 8 ml HNO_3_ + 2 ml H_2_O_2_, and 12 ml HNO_3_ + 3 ml H_2_O_2_, and further processed as described above. Membranes were then qualitatively examined for the presence of diatom fragments or incompletely digested tissue remnants via SEM.

Based on the related results (see the “SEM imaging” section), study case tissue samples of 0.5–1.0 g weight and 10 ml of reference liquids (drowning media/water controls) were equally treated with a 10 ml batch of the HNO_3_/H_2_O_2_ digestion medium.

### SEM analysis and image acquisition

After being attached to aluminum pin stubs with double-sided adhesive carbon tabs, membranes were sputter-coated with gold and analyzed in a Philips/FEI XL30 ESEM scanning electron microscope at 15 kV.

Peripheral tissues (liver and kidney) were qualitatively examined for the presence of diatoms by classification into one of the following categories: − (0 diatoms), + (1–4 diatoms), ++ (5–9 diatoms), or +++ (10 or more diatoms).

To quantitatively assess lung and water samples, the membranes were manually scanned at a magnification of 1000× to cover areas of appropriate size ensuring representative quantification while also allowing for easy identification of small diatoms.

**Fig. 2:**
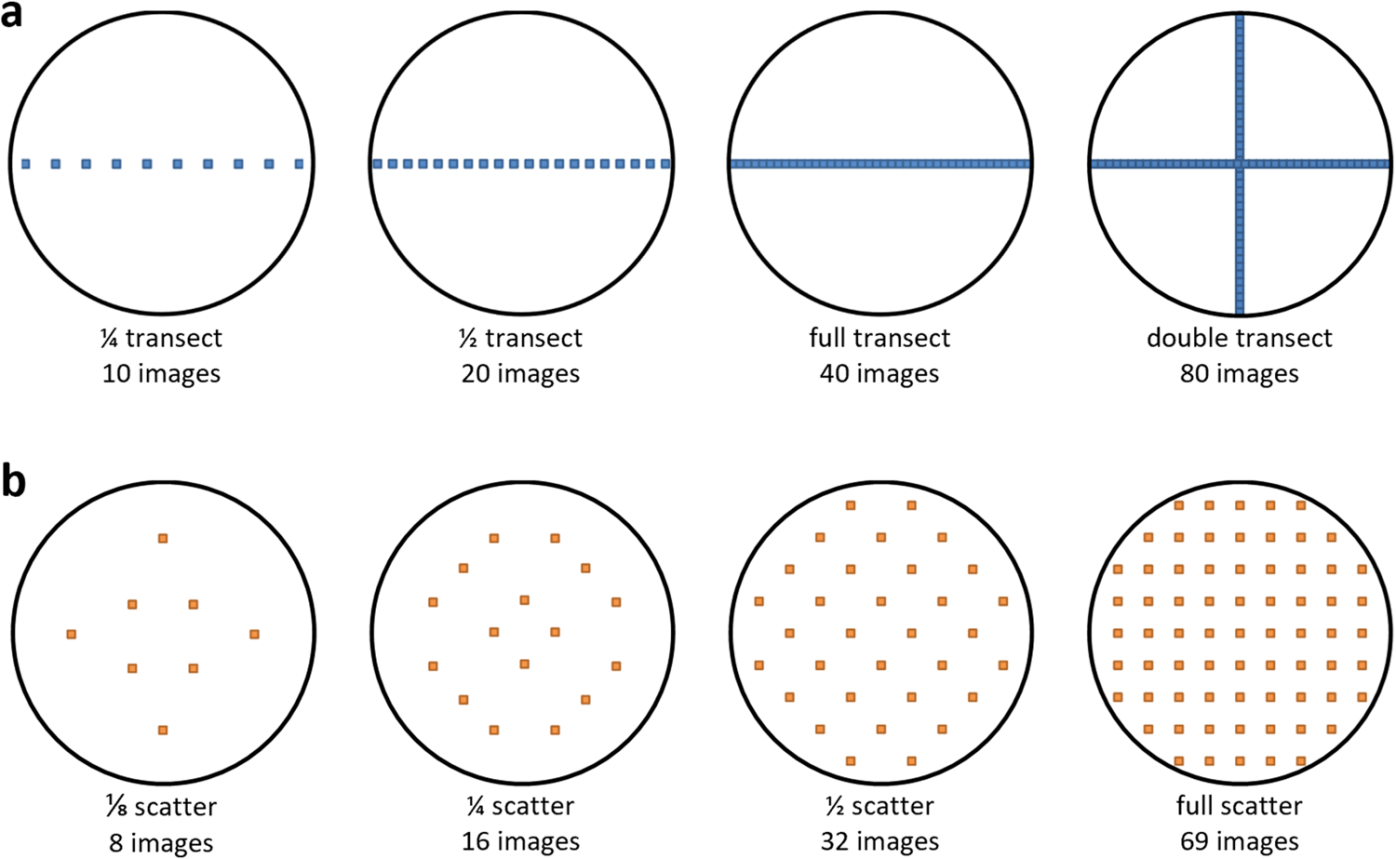
SEM imaging methods; **a **transectial image acquisition (quarter, half, full and double transects) and **b **scatter image acquisition (eight, quarter, half and full scatter)

Due to the limitation of non-automated (manual) SEM imaging, full coverage of the membranes would hardly be feasible as requiring more than 1700 images. Therefore, two alternative strategies with reduced imaging demand—transectial acquisition and scatter acquisition—were applied on diatom-rich water samples in three dilutions (1:1, 1:2, 1:5) and tested for their time requirement and capability to assess diatom abundance of ≥ 95 % accuracy. Transectial image acquisition was performed in line of the filter’s diameter as quarter, half, full, and double transect, whereas scatter image acquisition was performed as eighth, quarter, half, and full scatter at uniformly distributed coordinates (Fig. 2). Both strategies were evaluated concerning their efficiency (number of required images) to reach an extrapolation-accuracy of 95% to the total diatom count (i.e., double transects plus full scatter).

### Data processing and statistical analysis

Images of transectial and scatter image acquisition were quantitatively analyzed for diatoms by manual counting, using the cell counter tool of the ImageJ 1.53p software to keep track of the process and document the results. Individual counts were compiled to a total number of diatoms per filter, allowing to calculate diatoms per g values for tissue samples and diatoms per ml values for drowning media, which were then used to determine L/D ratios. Statistical analyses, including correlation analysis between diatom number and tissue weight, diatom count projection, and L/D calculations, were performed using Microsoft Excel and SPSS 27.0 software, regarding *p* < 0.05 as statistically significant.

## Results

### Digestive capability

**Fig. 3:**
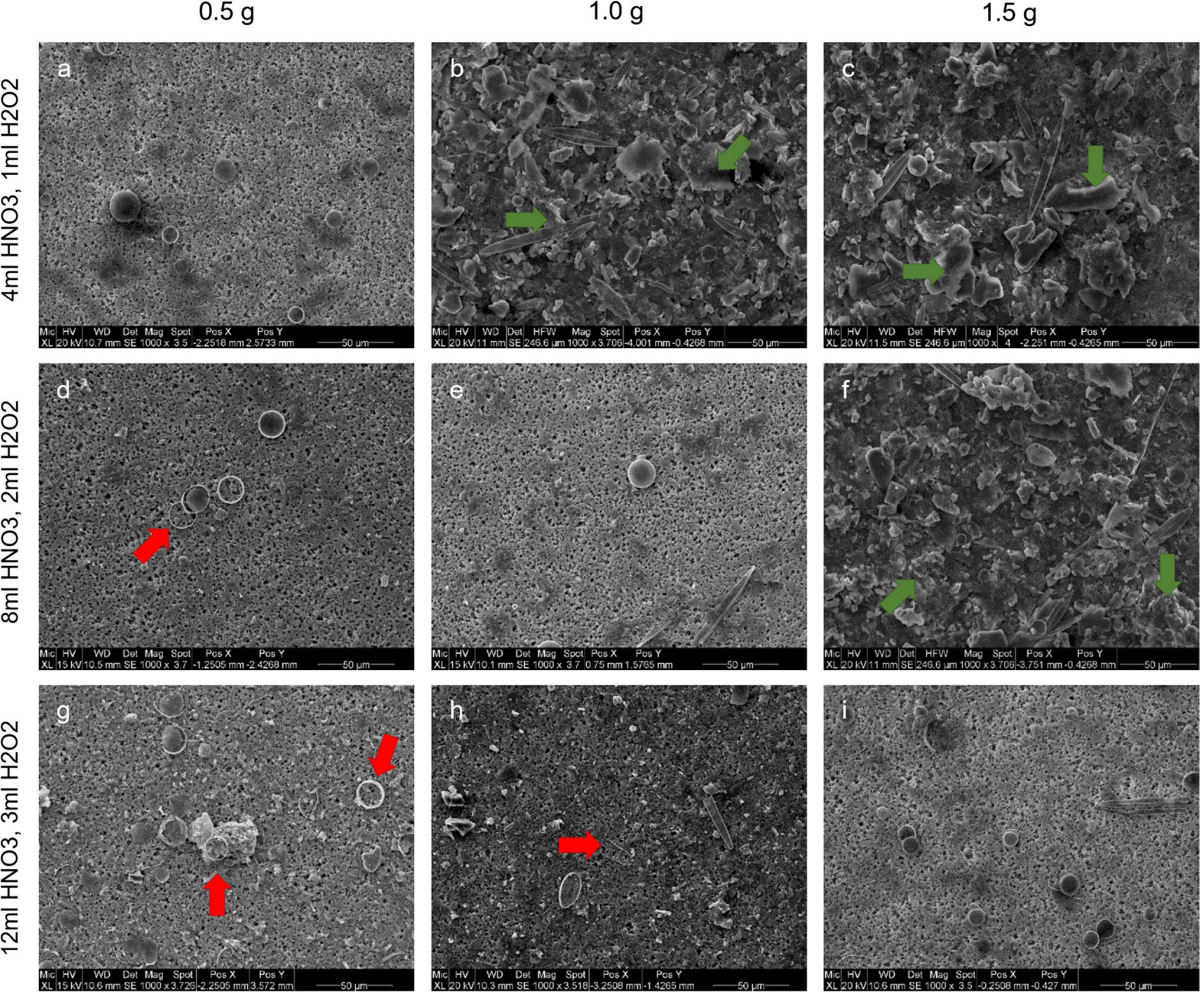
Digestive capability of different volumes of reagent: 0.5 g (**a, d, g**), 1.0 g (**b, e, h**), and 1.5 g (**c, f, i**) of lung tissue digested with 4 ml nitric acid and 1 ml hydrogen peroxide (**a, b, c**); 8 ml nitric acid and 2 ml hydrogen peroxide (**d**, **e**, **f**); and 12 ml nitric acid and 3 ml hydrogen peroxide (**g**, **h**, **i**); green arrows indicate organic remains (tissue residue); red arrows indicate partially disintegrated diatom frustules

Digestive capability tests, performed with different volumes of the HNO_3_/H_2_O_2_ digestion reactant on lung tissue samples of variable weight, showed the following results: The 5-ml batch (4 ml HNO_3_ + 1 ml H_2_O_2_) reached optimal digestion results with samples of 0.5 g (Fig. 3a) but resulted in partial (incomplete) digestion with samples of ≥1.0 g (Fig. 3b–c). By contrast, 10 ml of the reactant (8 ml HNO_3_ + 2 ml H_2_O_2_) was capable to completely dissolve samples of 0.5 g and 1.0 g (Fig. 3d–e) with slight diatom-dissolution at lower tissue weight (Fig. 3d), while tissue residues persisted with 1.5 g (Fig. 3f). Fifteen milliliters of the reagent (12 ml HNO_3_ + 3 ml H_2_O_2_) had a clearly higher digestive potential with 1.5 g samples (Fig. 3i) but promoted diatom disintegration in samples of lower weight (Fig. 3g–h). In addition, higher total volumes required longer filtration times, also potentially affecting diatom integrity.

### SEM imaging

**Fig. 4:**
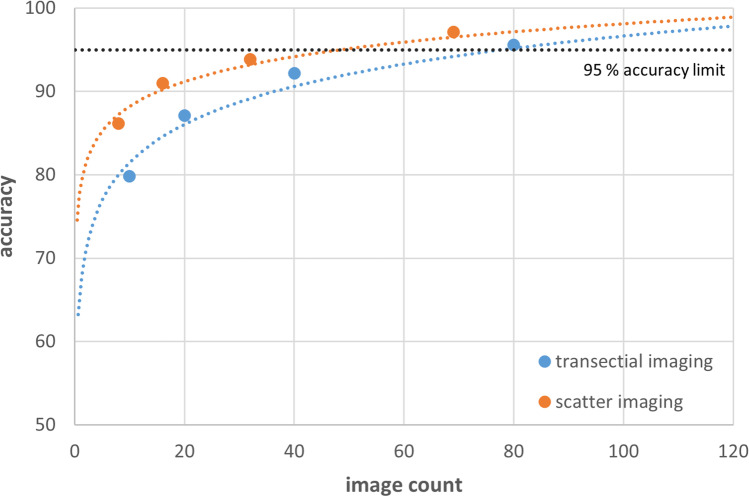
Accuracy of transectial image acquisition and scatter imaging compared with the number of images required to reach a 95% accuracy limit; formula of transectial imaging *y* = 6.5832ln(*x*) + 66.32; formula of scatter imaging *y* = 4.3587ln(*x*) + 77.99

Diatom abundances of each imaging strategy (transectial acquisition and scatter acquisition), initially evaluated in three dilutions (1:1, 1:2, 1:5) of diatom-rich control water samples and eventually combined for coherent accuracy assessment, yielded the following results: diatom count extrapolations from quarter transects (10 images), half transects (20 images), and full transects (40 images) achieved accuracy levels of 79.8%, 87.1%, and 92.2%, thus remaining below the required confidence limit of 95%. By contrast, the double transect method reached a value above the limit (95.6%) but required 80 images. Results from scatter acquisition proved distinctly different from those of transect acquisition. While the eighth scatter (8 images), quarter scatter (16 images) and half scatter (32 images) strategies (86.1%, 91.0%, and 93.8%) remained below the confidence limit as their counterparts in transect acquisition, the full scatter method achieved a value of 97% based on the analysis of only 69 images. Calculations to reach the 95% accuracy limit using the obtained logarithmic regression formulae resulted in theoretical thresholds of 78 images for transectial acquisition and 50 images for scatter acquisition (Fig. 4).

### Correlation between diatom quantity and tissue weight

**Fig. 5:**
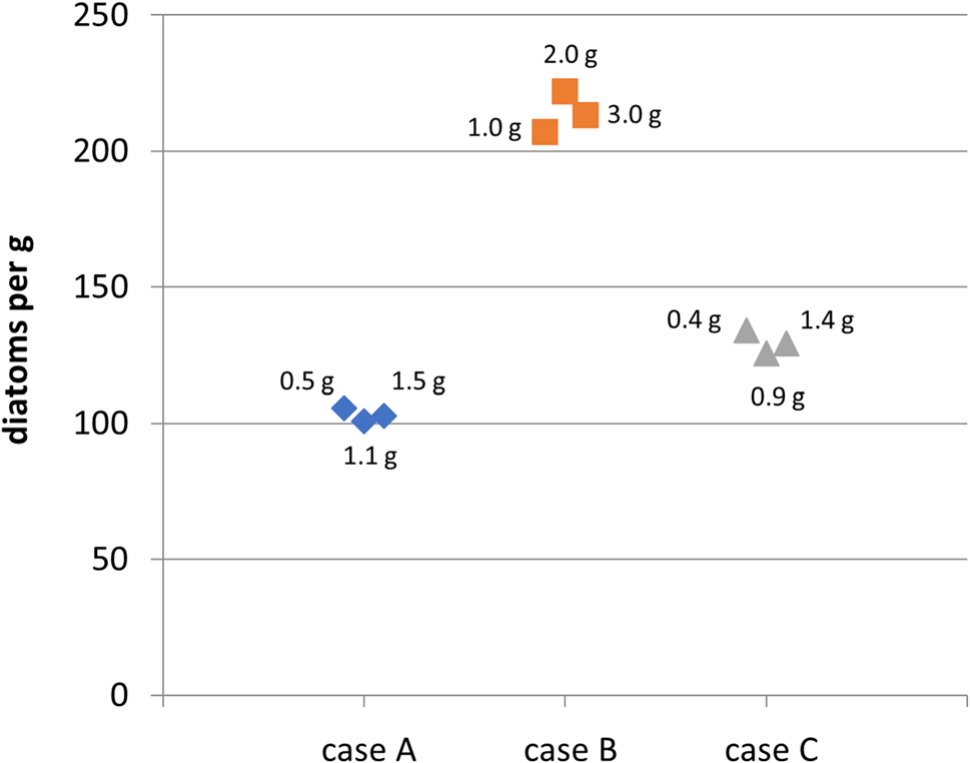
Correlation between lung tissue samples of different weight and their calculated diatom counts per gram in three control drowning cases

Correlation analyses results between diatom quantity and tissue mass (weight) of three control drowning cases (A, B, C) are summarized in Table 2. The Kruskal-Wallis comparison did not indicate significant differences between corresponding lung tissue samples of each case. Spearman’s correlation analysis showed a positive correlation between diatom number and tissue weight at high significance levels for all samples. Diatoms per gram calculations showed almost identical values for lung tissue samples within each respective case at a maximal deviation of 2.5% (Fig. 5).

**Table 2:** Statistical analysis and correlation of three lung tissue samples of different weight with their respective diatom counts in three different drowning cases

Control cases	A	B	C
Tissue weights	0.5/1.1/1.5	1.0/2.0/3.0	0.4/0.9/1.4
Number of diatoms	57/108/155	207/444/640	55/108/181
The Kruskal-Wallis test	*p* = 0.149	*p* = 0.229	*p* = 0.841
Spearman’s correlation	*r* = 0.406 *p* = 0.000	*r* = 0.681 *p* = 0.000	*r* = 0.573 *p* = 0.000

### Case analysis applying L/D ratios

**Fig. 6:**
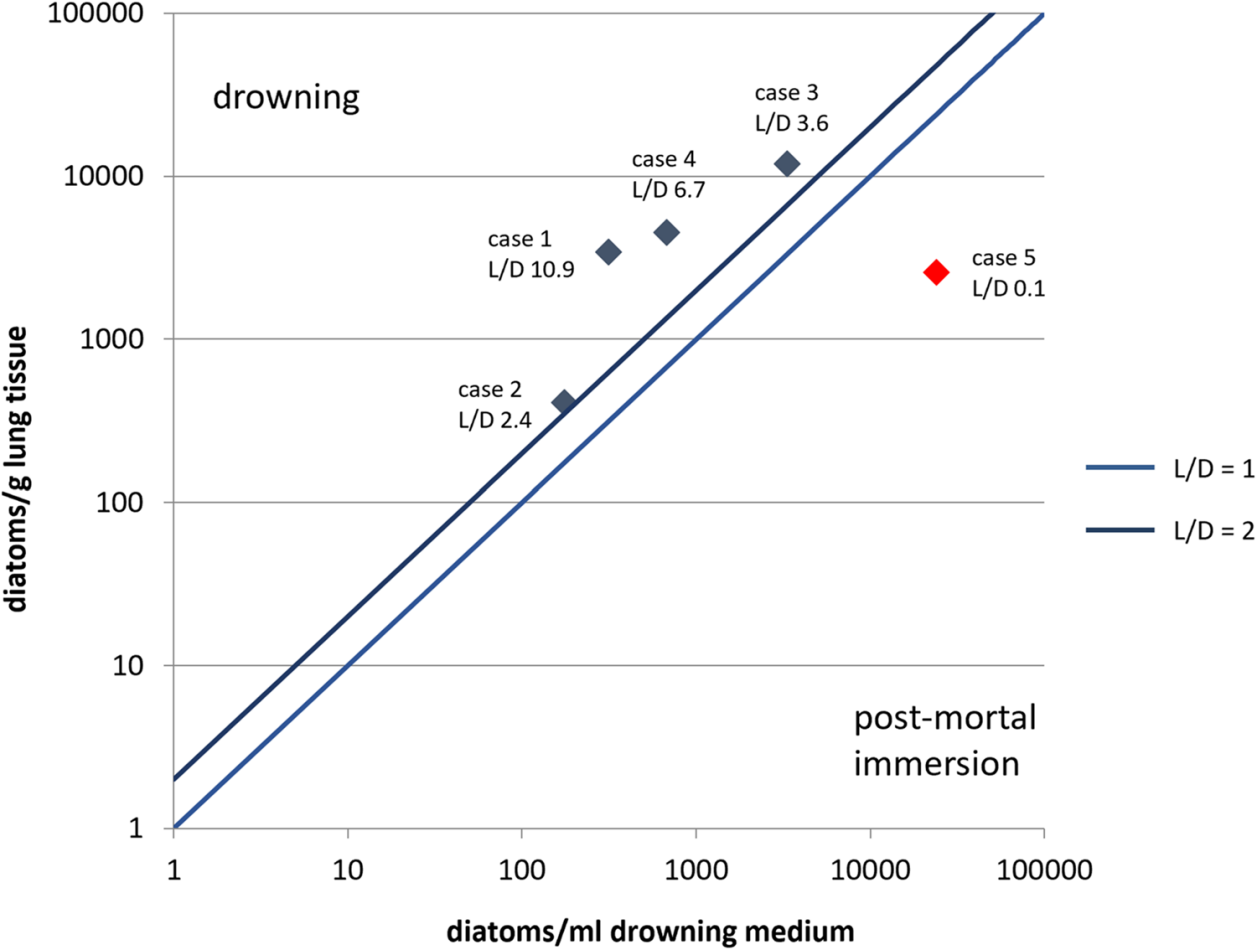
L/D ratios of cases 1–5 on logarithmic scales of diatom count in lung tissue and drowning medium. L/D values >1 assume drowning, L/D values <1 assume post-mortal immersion

All investigated drowning media and lung tissue samples contained diatoms in quantities sufficient for analytical application. Peripheral tissue analysis (qualitative SEM analysis) revealed diatom presence in all kidney samples, as well as in liver tissue samples from cases 1, 4, and 5 (Supplements 2). All cases showed species conformance between tissues and drowning media. Except for case 5, all lung tissue samples displayed higher diatom numbers than the corresponding drowning media, consequently resulting in L/D ratios above 1, even exceeding a value of 2 (Fig. 6), thus showing that the diatom concentration in lung tissue was at least twice as high than in the corresponding drowning medium. By contrast, case 5—although displaying a relatively high diatom number per gram of lung tissue—reached an L/D ratio of 0.1, as the concentration of diatoms per ml drowning medium reached a value almost ten times higher than in the lung (Table 3).

**Table 3:** Diatoms per gram lung tissue and per ml drowning medium and calculated L/D ratios of cases 1–5

Case no.	Diatoms per g lung tissue	Diatoms per ml drowning medium	L/D ratio
1	3426	315	10.9
2	410	175	2.3
3	11928	3336	3.6
4	4521	675	6.7
5	2567	24148	0.1

## Discussion

The diatom test has long been a controversial technique in forensic drowning diagnosis, most prominently due to its sensitivity to contamination and risk of incorrect results even in qualitative analysis [5, 9–12]. In recent years, an increasing interest of quantitative diatom testing has promoted re-evaluation of the approach [4, 13–15]. Especially the comparison of diatom content in lung tissue and drowning medium (L/D value) has changed the significance of the method [8]. Rather than utilizing timesaving and less expensive light microscopy, the preference of SEM application empowered diatom-based forensic drowning diagnosis providing much higher resolution and the options for automated scanning and diatom counting [16, 17], as well as species identification by artificial intelligence [18–20]. However, to date the method is only applied in a limited number of institutes, not least because of its technical requirements. Results of the present work enabled the establishment of a protocol for quantitative diatom analysis using/requiring comparably limited resources.

### Notes on methodology

To warrant the accuracy of the modified method for SEM-based forensic diatom testing, some critical aspects have to be considered:

(i) All reagents and supportive materials need to be validated for diatom absence. This is of particular relevance as diatomaceous earth is a commonly used filtration aid, traces of which may remain in chemicals [21]. Compared to qualitative investigation, these traces may be deemed marginal in quantitative approaches [15, 22], but are of special importance for bodies of water with low diatom concentrations.
(ii) Diatom treatment (protocols) should have the capability to assure complete dissolution of all organic matter while leaving diatom frustule integrity intact. It appears optimal to digest tissue samples at around 1.0 g with 10 ml of a digestion medium composed of 8 ml HNO_3_ and 2 ml H_2_O_2_, whereas samples of 0.5 g or less may also be sufficiently digested at smaller volume of the digestion reagent. Analogously, tissue samples over 1.0 g in weight should be treated with a digestive volume larger than 10 ml to reach satisfactory results, which clearly confirmed that the volume of digestion media applied to a certain amount of tissue can substantially influence the quality of the performance, regardless of whether quantification is carried out automated or manually.
(iii) In this context, the present work specified the necessity to assess the replicability of the methods outcome. We could show that, regardless of the applied tissue mass, calculated diatom concentrations per gram are highly stable. This seems especially important when membranes clog during filtration and/or when the initial analysis results in very high (diatoms in clusters or layers) or very low diatom numbers on the filter. In such cases, it may be necessary to modify (enlarge or reduce) tissue sample volumes in order to ensure a reliable analysis of the diatom number.
(iv) Another crucial aspect for a wider practical application is to optimize the method`s cost and effort without negatively influencing the diagnostic quality. In this respect, a major task was to determine the minimum number of required images to obtain representative data for the entire membrane, irrespective of whether dissolved tissue or diatom-containing water (drowning media) are examined. Experimental conditions revealed that regular diatom distribution on the membrane is not granted. An evaluation of 10 equidistantly scattered images over the filter resulted in a mean error of 12%. This error accumulated to 19% when the same number of images were taken equidistantly along a transect, clearly suggesting that scatter imaging should be preferred over transectial acquisition. Our data indicate that a total of 50 equidistant scatter images distributed over the entire filter should be sufficient to produce reliable results beyond the 95% probability limit.

### Significance in practical application

Despite the small sample size, the present trial conducted on five autopsy cases indicates that the modified method of SEM-based diatom testing has indeed true application value in forensic drowning diagnosis.

Previous studies reported drowning probabilities of 96% for L/D values >1 and even 100% for L/D values >2, while yet evincing limited conclusiveness between drowning and post-mortal immersion at values ≤1 [8]. Investigation of the cases 1–3, which all presented distinct classical drowning signs [23, 24], achieved L/D values between 2.4 and 10.9, which therefore can be considered strong evidence for drowning [25–27].

Case 4 showed insufficient evidence to be diagnosed as drowning case based on classical drowning signs, most likely due to advanced decomposition. However, the determined L/D value of 6.7 confirmed drowning as the cause of death, as a 6.7-fold diatom concentration in the lung compared to the drowning medium is very difficult to explain by other mechanisms than active aspiration and pressure filtration of the drowning medium in the lung [22, 28, 29]. This case underlines the diagnostic potential of the modified SEM-based diatom test in cases of advanced decomposition which frequently lack other drowning signs [30].

Case 5, although exhibiting several classical drowning signs, only reached an L/D value of 0.1, rather indicating post-mortal immersion than drowning. However, specific circumstances of this case leave some caveats. The collected drowning medium contained plenty of debris from aquatic plants, which is most likely the cause for the very high number of diatoms detected (>24.000 diatoms per ml), as diatoms are known to colonize on aquatic plants epiphytically [31]. It must be questioned whether the secured materials represent the actual diatom concentration of the drowning medium at the time the body got into the water. Perhaps, actively aspired water contained less diatoms as suggested by the present analysis, consequently the debris would have had a major effect on the L/D value and the interpretation of the result. This depicts the limitation, that methods of SEM-based diatom testing are inherently dependent on the reliability of the secured drowning medium. Consequently, sampling requires special caution to not distract the water body’s diatom homogeneity. Other factors potentially affecting this reliability include sampling from wrong depth or location [32] and delayed sampling, allowing for diatom concentration changes in the waterbody [26, 33]., e.g., after heavy weather conditions [34]. In this relation, additional research providing reference data on species and abundances from spatial, temporal, and seasonal diatom mapping of local natural waters, as already implemented in some countries, could greatly improve the validation of drowning media [35, 36].

## Conclusion

With some adaptions in sample processing and SEM imaging, we were able to apply a new setup of quantitative diatom assessment at our institute. Our data show that the modified method of SEM-based diatom testing has high potential to become a standard technique in forensic drowning investigation, particularly in cases of advanced decomposition, despite the necessity to critically consider the limitations of the application and outcome interpretation.

## Supplementary Information

ESM 1: Supplements (PDF 1287 kb)

## Data Availability

All data generated or analyzed in course of this study are included in the article.
