# Supplementary material for: Validation and optimization of the diatom L/D ratio as a diagnostic marker for drowning
Source: Int J Legal Med. 2023 Mar 4;137(3):939–48. doi: 10.1007/s00414-023-02970-x (PMC10085902; doi:10.1007/s00414-023-02970-x)
Supplement: Supplementary file 1 — Supplements (PDF 1287 kb) [file 414_2023_2970_MOESM1_ESM.pdf]

Supplements

1. SEM control images

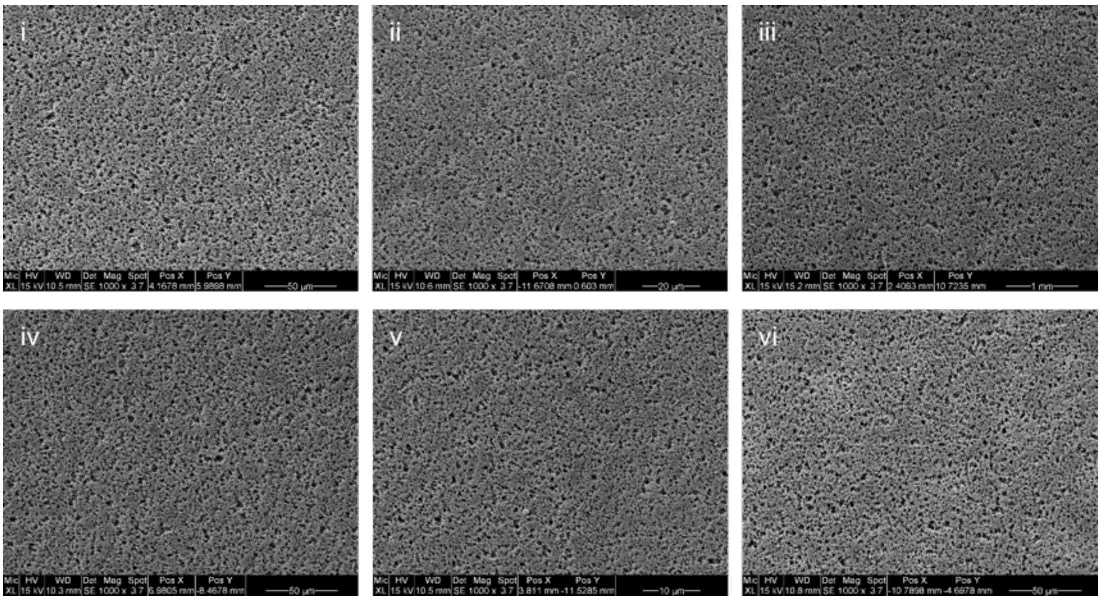

Figure S1 Exemplary negative control images for diatom-content in working reagents; **i** nitric acid, **ii** hydrogen peroxide, **iii** ultrapure water, **iv** ethanol, **v** tap water, **vi** digestion vapor

2. Qualitative SEM analysis of peripheral tissue

Table S1 Qualitative SEM-analysis for diatoms in liver- and kidney tissue of case 1-5; **(-)** indicates zero diatoms, **(+)** indicates ≥ 1 diatoms, **(++)** indicates ≥ 5 diatoms, **(+++)** indicates ≥ 10 diatoms

case no.	liver	kidney
1	++	+++
2	-	++
3	-	+
4	++	++
5	+++	+++

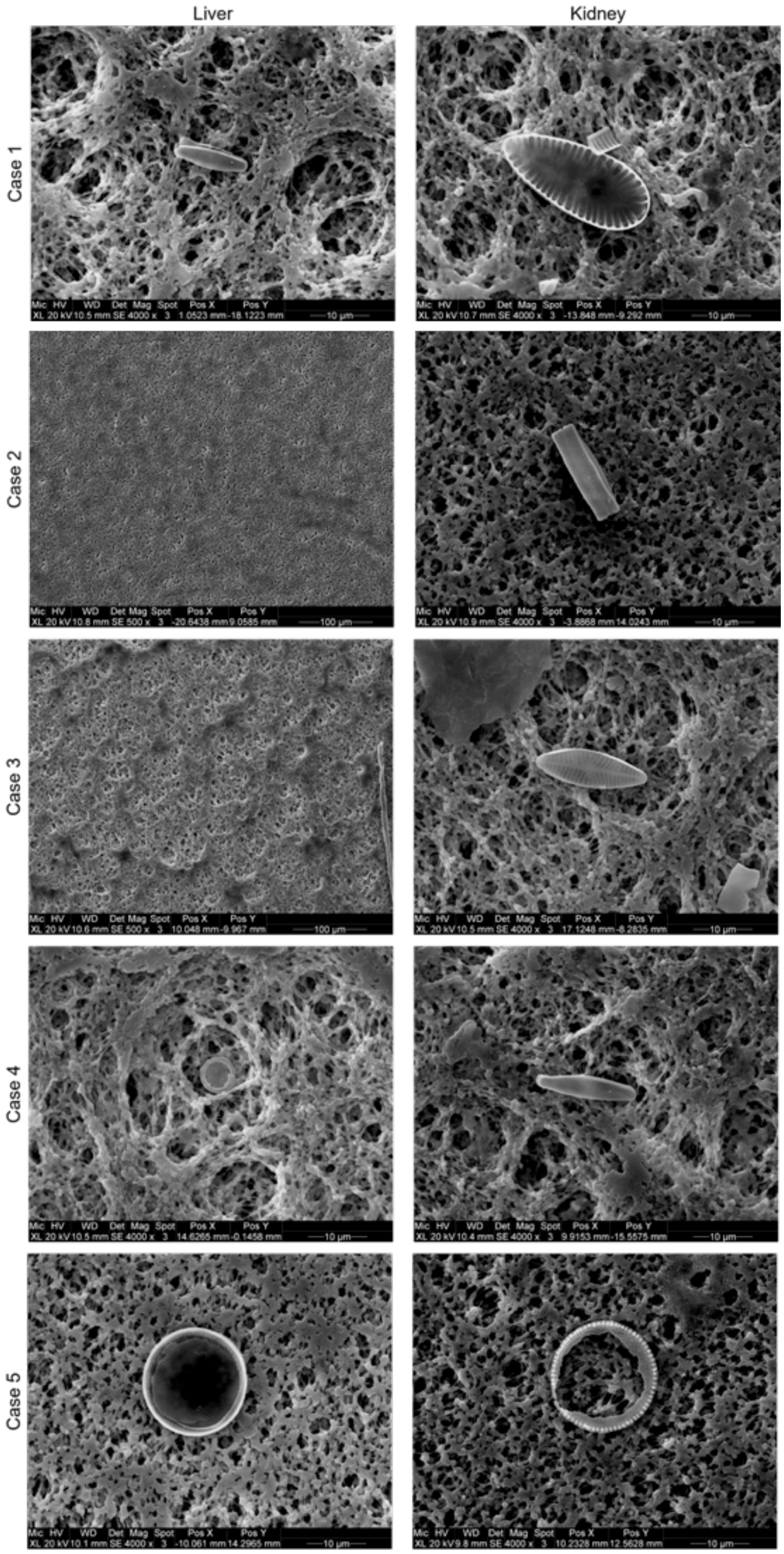

Figure S2 Exemplary images of diatoms in liver- and kidney tissue of cases 1-5.
